# Self-assembling peptide scaffolds in the clinic

**DOI:** 10.1038/s41536-020-00116-w

**Published:** 2021-02-17

**Authors:** Fabrizio Gelain, Zhongli Luo, Marika Rioult, Shuguang Zhang

**Affiliations:** 1 Institute for Stem-Cell Biology, Regenerative Medicine and Innovative Therapies (ISBREMIT), IRCCS Casa Sollievo della Sofferenza, 71013 San Giovanni Rotondo, Italy; 2 Center for Nanomedicine and Tissue Engineering (CNTE), ASST Grande Ospedale Metropolitano Niguarda, 20162 Milan, Italy; 3 College of Basic Medical Sciences, Molecular Medicine and Cancer Research Centre, Chongqing Medical University, Chongqing, 400016 China; 4 3-D Matrix Inc., Needham, MA 02494 USA; 5 Laboratory of Molecular Architecture, Media Lab, Massachusetts Institute of Technology, Cambridge, MA 02139-4307 USA

**Keywords:** Regenerative medicine, Translational research

## Abstract

Well-defined scaffold hydrogels made of self-assembling peptides have found their way into clinical products. By examining the properties and applications of two self-assembling peptides—EAK16 and RADA16—we highlight the potential for translating designer biological scaffolds into commercial products.

## Introduction

Self-assembling peptide (SAP) scaffolds can be traced back to the fundamental discoveries made in 1951 by Pauling et al., who reported the molecular structures of the most fundamental motifs of proteins, namely the alpha-helix^1^ and the beta-sheet^2^. The beta-sheet structure is the molecular basis of most SAP nanofiber scaffolds. However, it was not until the serendipitous discovery of a SAP as a repeating segment in the yeast protein *Zuotin*^3^ in 1990 that the concept of peptides as well-defined nanomaterials was recognized. Since then, peptide nanofiber scaffolds have been designed as 3D scaffolds for the serum-free culturing of tissue and stem cells, for reparative and regenerative medicine and for accelerated wound healing and surgery. In this perspective, we examine the translational advances and commercialization of SAP-based nanomaterials by highlighting the properties and design of two peptides that are used in clinical applications, that is, EAK16- and RADA16-derived SAPs. Numerous other non-SAP hydrogels have been used in various clinical and preclinical applications in last few decades. Readers who are interested in other nonpeptide biomaterials for clinical uses should consult the extensive literature^4–8^.

The translation of SAP nanofiber scaffolds into commercial products requires: (1) they provide a functional improvement over existing products on the market; (2) they are marketable at a cost similar or lower than the competing products; (3) they can be scaled up at a reasonable cost; (4) they are completely harmless to animal and human tissues; (5) they are beneficial for medical and societal needs; and (6) they are approved by appropriate regulatory agencies for widespread use.

## Designing peptide nanofiber building blocks

The first SAP, EAK16, was unexpectedly discovered as an unusual repetitious segment (AEAEAKAKAEAEAKAK) in a natural yeast protein^3^. To mimic the self-assembly properties of this naturally occurring SAP, two variants, RADA16-II^9^ and RADA16-I^10^, were synthesized using natural L-amino acids and solid phase chemistry. These sequences were inspired by the almost ubiquitous presence of RGD (arginine, glycine, aspartic acid) as a functional motif from fibronectin and other extracellular matrix proteins, stimulating adhesion and survival of cultured cells in vitro^11^, where A (alanine) was chosen instead of G (glycine) to reduce flexibility of the peptide chain and warrant appropriate stability of the self-assembled nanofibers.

**Fig. 1:**
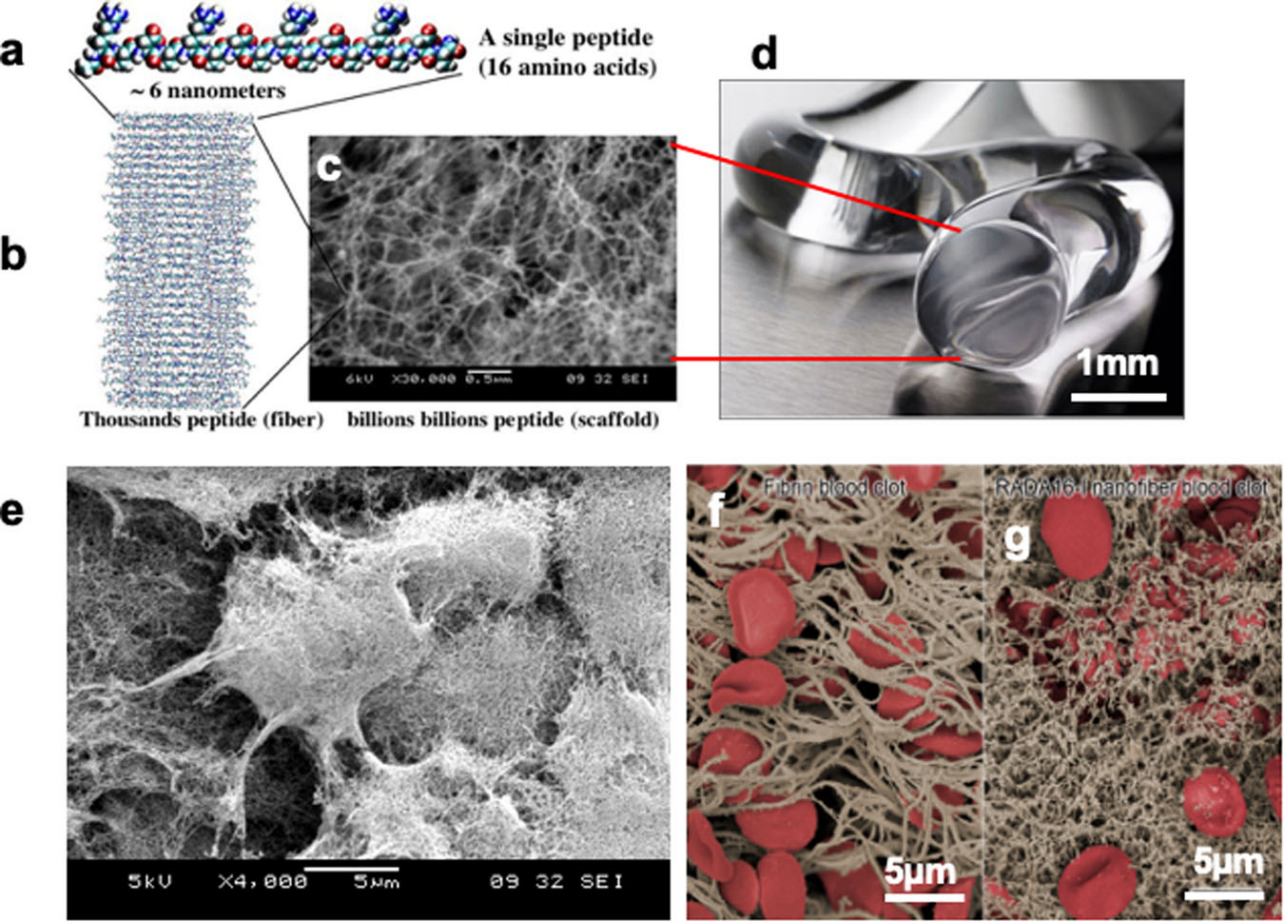
Beta-sheet self-assembling peptide nanofiber scaffolds, hydrogel and cells embedded in the scaffold. **a** Self-assembling peptide RADA16 molecular model. The calculated peptide dimensions are ~6 nm long depending on end capping, 1.3 nm wide and 0.8 nm thick. **b** Model of thousands of individual peptides as a single nanofiber. **c** Trilion of individual peptides self-assemble into nanofiber scaffold as revealed by the SEM. Scale bar = 0.5 µm. **d** RADA16-I peptide nanofibers scaffold hydrogel with extremely high-water content (99.5–99.0%, w/v water). An image of nanofiber scaffolds hydrogel of RADA16-I at 1% (10 mg/ml), 99% water (image courtesy of 3-D Matrix). **e** Murine neural stem cells are embedded in the nanofiber scaffold. The diameter of nanofiber is in the similar diameter as the cell produced extracellular matrix^8^. **f** Red blood cells are embedded in the natural fibrin. **g** Dense RADA16 nanofiber scaffold^29^. Billions of layers of interwoven nanofibers tightly keep blood cells in place thus preventing from bleeding.

The D-form amino acids were used for the design of chiral D-form SAPs, because chiral D-peptides are resistant to L-protease degradation^12^, enabling their application in vivo. In general, the structure of SAPs can be regulated by the amino acid sequence, the peptide concentration, pH, temperature, and the ionic composition of the medium. Fine-tuning of these conditions allows the design of dynamic peptide structures as well as their self-assembly process and formation of nanostructures (Fig. 1).

## SAP nanofiber scaffolds for cell repair and regeneration

SAPs fulfill many of the engineering requirements for tissue scaffolds, in particular, biomimetic tunability^13^. The mechanical tunability and 3D spatial organization of SAPs make them ideal building blocks of scaffolds for in vitro 3D tissue cell culture, in which they can serve as soft fillers embedded in stiffer synthetic biocompatible biopolymers. In general, to be useful for tissue engineering, SAP scaffolds have to mimic the biomechanics of the specific tissue type, display multiple functional motifs to allow interaction with cells and/or cytokines involved in the regenerative process, have a bioabsorption time compatible with tissue regeneration kinetics, display nanostructures and porosity to facilitate tissue infiltration and cytokine diffusion, and self-assemble in response to a stimulus compatible with other biological scaffolds.

Owing to the ease of biomimetic functionalization, their nanostructured morphology, their mechanical tunability, their high content of water or medium (~99%), their permeability, and, most importantly, their excellent transparency, SAPs are becoming an important synthetic microenvironment for cell^14^ and stem cell differentiation in vitro^14–16^. To achieve the required mechanical properties favoring cell culturing^17^, transplant engraftment and tissue regeneration SAPs: (1) can be dissolved at different concentrations (e.g., from 1 to 5% v/w)^16^; (2) can be mixed with multibranched SAPs sharing the same self-assembling backbones^18^; (3) can be cross-linked with either natural^19^ or synthetic^20^ cross-linkers; and (4) lastly, they can be point-mutated at their sequence, altering the overall hydrophobic/hydrophilic balance^21,22^.

In terms of biomimetic functionalization, RGD and BMHP motifs, that is, cell instructive cues, can be linked to RADA16-I^14^ to trigger proliferation, differentiation, and neurite outgrowth of neural stem cells in serum-free 3D systems^14,16^. Double functionalization of RADA16-I with both RGD and IKVAV can improve viability and differentiation of neural stem cells over only one function^23^.

Similarly, (LDLK)_3_ functionalized with the phage display-derived functional motifs KLPGWSG and FAQRVPP triggers neuronal and oligodendroglial differentiation better than in standard culture conditions (Cultrex BME)^24,25^. The addition of plain (LDLK)_3_ refines the composition of a multifunctionalized (LDLK)_3_-based SAP. When mixed with branched (LDLK)_3_^18^_,_ the mechanical stiffness can be optimized to allow the formation of a dense 3D network of differentiated and electrically active mature neurons, astrocytes, and oligodendrocytes in long-term cultures. GABAergic, glutamatergic, and cholinergic neurons have been successfully cultured in SAP scaffolds. In addition, cells expressing basic myelin proteins in serum-free cell cultures can be differentiated from human good manufacture practices (GMP)-grade neural stem cells in SAP scaffolds, which can potentially be used as patches or microchannels for spinal cord injury regeneration and/or for in vitro models of nervous tissue^26,27^. Neural stem cells encapsulated in a functionalized RADA16-IKVAV nanofiber scaffold are applicable for rat brain injury repair. The RADA16-IKVAV material not only improves the survival of encapsulated neural stem cells, but also reduces the undesired glial astrocytes^28^. The RADA16 scaffold also supports epithelial morphogenesis^13^ and improves osteogenic differentiation^29,30^, cardiac progenitor cells^31^, and clinical-grade bioartificial liver functions including detoxification and biotransformation^32^.

**Fig. 2:**
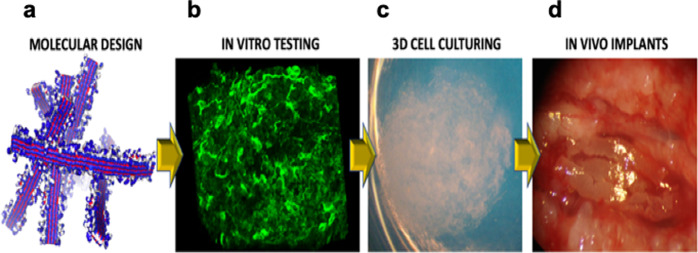
The assembly line of self-assembling peptides toward clinics. **a** Molecular modeling for both all-atom and coarse-grained simulations of self-assembling peptides featuring different functionalizations are necessary to verify the self-assembling propensity and solubility of new sequences^24^. **b** Nanofibers of self-assembling peptides scaffold hydrogels are subsequently tested with cells (either to be transplanted or from the target tissue) in vitro to verify cytotoxicity and biomimetic properties of the novel construct. **c** Synthetic self-assembling peptides are tailored into 3D constructs with densely seeded cells (image courtesy of Amanda Marchini), potentially leading to organoids in long-term cultures in serum-free conditions. **d** Selected self-assembling hydrogels (seeded with cells or loaded with drugs) are injected or positioned (after self-assembling) in in vivo animal models of the target pathology. All steps provide essential data for subsequent clinical approval.

Nervous tissue cells are highly sensitive to the mechanical properties and bioactivity of the surrounding microenvironments. The stiffness of nervous tissue (100–1000 Pa)^17,33,34^ is similar to that achievable with SAP nanofiber scaffolds. Moreover, SAPs can be functionalized with laminin moieties, which is a key protein in the extracellular matrix of brain tissue^14,28,35^. Therefore, SAP scaffolds offer ideal properties for the culture of nervous tissue. SAP scaffolds can also match the flexibility and compliance of peripheral nerves as well as the 3D spatial cytoarchitecture of the central nervous, which are a key to successful nerve regeneration in vitro^19,20^. For example, nervous tissue regeneration and functional recovery can be achieved using RADA16-I functionalized with the BMHP1 active motif, preassembled RADA16-I, and LDLK12 seeded and precultured in vitro with NSCs^14,26,27^. Then, the functionalized RADA16-I is loaded with a mix of proregenerative cytokines and injected into electrospun microchannels (Fig. 2). Diluted SAPs (with the lowest G′) allow diffusion within acutely injured spinal cord tissue without perturbing the preexisting neural circuitry, attenuate hematoma, and foster an increased number of regenerated axons, leading to the formation of electrically active neuronal networks^36,37^.

Similarly, SAP scaffolds can be used to promote wound healing after surgical procedures, traumatic injury, chronic diabetic ulcers, and bedsores. SAP scaffolds do not elicit any noticeable adverse immune responses or inflammatory reactions, as they are made of pure synthetic amino acid without animal-derived components or chemical, biological, or toxic contaminants. Therefore, SAP scaffolds have been approved for wound healing applications in humans, with additional trials for several indications already planned or launched for human tooth wound healing and skin wound healing^38^. Chiral SAP scaffolds can further incorporate components for disinfecting and protecting superficial wounds and abrasions (Chuan Rong, approval number: 20190063, Sichuan, China).

## Rada16 in surgical use

**Fig. 3:**
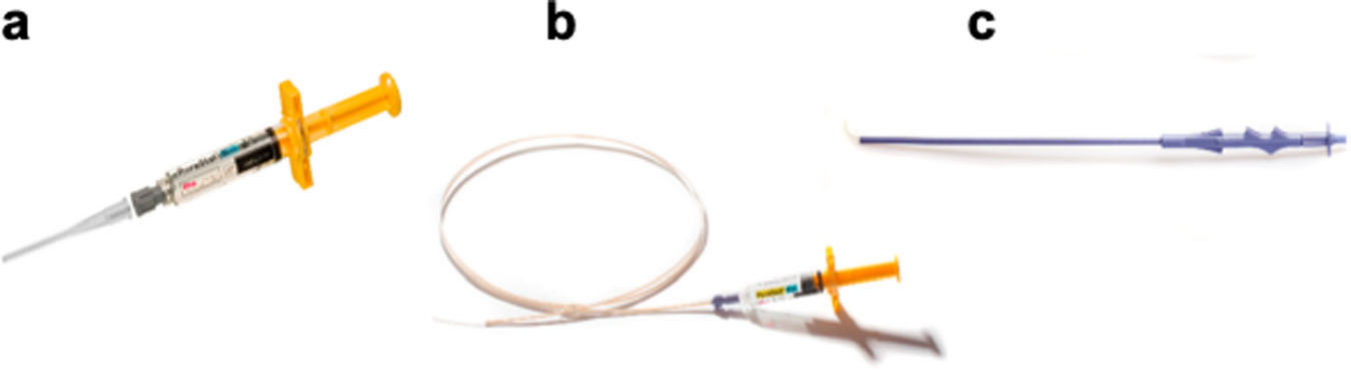
PuraStat^®^ made of self-assembling peptide nanofiber scaffolds and PuraStat^®^ accessories. **a** 5 ml syringe with attached nozzle applicator, ready for use as a hemostatic agent during surgery after opening of the packaging and attachment of the nozzle applicator. **b** Endoscopic nozzle (160 mm) attached to a PuraStat^®^ syringe. **c** Laparoscopic nozzle.

The SAP RADA16 (Fig. 1) is sold as a hemostatic agent for surgery (PuraStat^®^ (2.5% w/v aqueous solution) and as a reagent for life science research, PuraMatrix^®^ (1% solution) (Fig. 3). PuraMatrix^®^ is sold directly by 3-D Matrix and through distributors including Corning, VWR, Fisher Scientific, and others. PuraStat^®^ is a CE-marked class III medical device for hemostasis during surgery, especially for bleeding from small blood vessels and oozing from capillaries of the parenchyma of solid organs as well as oozing from vascular anastomoses. PuraStat^®^ is also indicated for the reduction of delayed bleeding following gastrointestinal endoscopic submucosal dissection in the colon. No contraindications have been identified so far. PuraStat^®^ is sold in Europe, Australia, New Zealand, Southeast Asia, the Middle East, and Latin America and it has been approved by Health Canada. Scaled-up industrial production of PuraStat^®^ and other 3D scaffolds or sphere forming SAPs demands the yield of consistently high-quality products at affordable costs. 3-D Matrix and partners also currently carry out clinical trials for SAP scaffolds for drug release to treat triple-negative breast cancer (Japan, UMIN000016790), in tissue regeneration for dental sinus lift procedures (US, IDE0100253), and preclinical trial in surgery to remove cancerous tissue from the digestive tract (Paris, France, no trial number).

## Chiral D-form SAP scaffolds

**Fig. 4:**
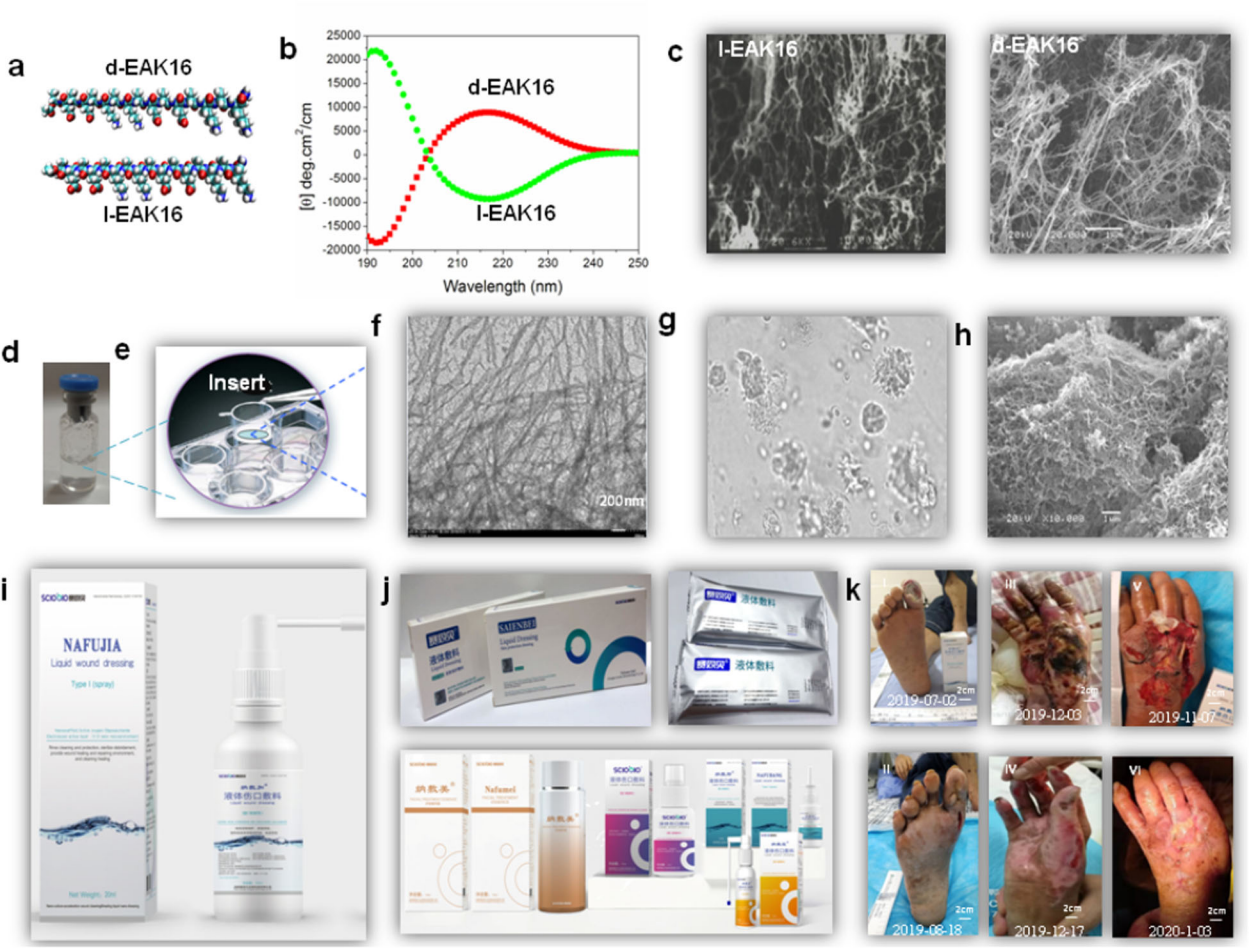
The chiral self-assembly D- and L-peptide system. **a** Molecular models of d-EAK16 and l-EAK16. **b** CD spectra of d-EAK16 and l-EAK16 at 37 °C. **c** SEM photograph of l-EAK16 and d-EAK16. **d** The nanofiber scaffolds of the chiral self-assembling peptide (including Sciobio-II). **e** The nanofiber scaffolds used in 3D cell cultures. **f** The TEM images show the nanofiber scaffolds. **g** 3D cell culture as an ECM viewed by microscope. **h** SEM image of surface of cells covered by enclosed nanofibers. **i** Sciobio^®^ commercial products sold as prefilled, sterile chiral self-assembling peptides to hydrocolloid solution as Sciobio liquid wound dressing, used in cleaning, washing, and protecting superficial wounds, abrasions in the superficial dermis. It forms a protective layer and acts as 3D nanofiber scaffold with microenvironment to promote wound healing. **j** Other chiral self-assembling peptides (Sciobio^®^, Saienbei, Nafumei, Nafujia, Nafubao, Nafubang) as cosmetics, facial mask, moisturizing lotion, eye cream, and tonic, or used in wound healing, tissue regeneration, and tissue cell and stem cell encapsulation. Updated information is available at www.sciobio.com (for images of **i** and **j:** reproduced by permission of Chengdu Sciobio Biotech, Co. Ltd, China, Copyright 2020). **k** The result to use Nafujia wound liquid dressing treat patients with chronic diabetic foot ulcer (I, before treatment, II, 47 days after, treated by Dr. Xia Yuping, international stoma therapist of vascular surgery, the First People’s Hospital, Yibin city, China); use Saienbei liquid dressing treat patients with deep second degree burn (III, before treatment, IV, 14 days after, treated by Dr. Zeng Shengqiang, TMC Hospital affiliated to Southwest Medical University); and use Nafujia and Saienbei liquid dressing treat patients with severe trauma (V before treatment, VI, 60 days after, treated by Dr. Wang Xiuying, Shexian Hospital, Hebei province).

Chiral self-assembling SAPs were designed using D-amino acids or a combination of alternating D- and L-amino acids^6^, such as d-EAK16. However, the secondary structure of d-EAK16 is less stable than that of l-EAK16. The stability can be tailored by adjusting the temperature, pH, and salts. Nanofiber scaffolds made of d-EAK16 are hydrated and contain >99% water^12,39,40^. Several chiral SAPs have been produced as commercial products, including Sciobio-I, -II, -III, and -IV (Fig. 4). These scaffolds are approved for use in accelerated wound healing, uterine repair, myocardial infarction repair, cartilage repair, and bone repair and have been used to treat patients with severe bedsore and chronic diabetic ulcer.

SAPs have been accidentally discovered, and have since been translated into a variety of clinically applicable products. Key to the successful and rapid translation of SAPs was the focus on clinical needs and commercialization, which can be achieved by focusing on regulatory questions and commercialization requirements early in the research process.

## Other SAP scaffolds in clinical trials

Other SAP-based products have been tested in clinical trials and are already available on the clinical market in recent years. Among them, a clinical safety trial was conducted with P_11_-4 SAP in the treatment of early dental care lesions^41^, showing negligible adverse effects not related to the treatment and a significant enamel biomineralization within the lesion cavities. Later on, in a prospective, randomized clinical trial, the same SAP was demonstrated to be effective, and significantly better than standard treatments, in regenerating decayed enamel, favoring the formation of novel hydroxyapatite at the bottom of early carious lesions^42^. Recently, another SAP, named T45K, was tested for safety and efficacy as hemostat solution for small-size skin wounds in a blind clinical trial^43^. Authors showed that with T45K, if compared to control treatment, a faster time to hemostasis and superior wound healing could be achieved also in patients undergoing antiplatelet therapy. On the other hand, safety profiles were similar to control. The lack of adverse effects in all clinical trials of SAPs seems to point out the promising potential and safety of such biomaterials in clinics.

## Rada-like SAPs vs other SAPs

The field of self-assembling is rapidly expanding, and a number of alternative sequences have been developed, yielding to heterogenous self-assembled nanostructures such as nanovescicles, nanorods, nanotubes, or nanoribbons. For a detailed description of other SAP-based therapies not yet into clinical testing, we link the reader to dedicated comprehensive reviews^44–46^ while we focus our attention on peculiarities of RADA16/LDLK12/EAK12 in respect to other widely published SAPs. RADA16 molecules can be easily functionalized at one or both termini by simply extending their backbone sequence and interspacing it with a pool of Gly: this process, with some limits^47^, allow a considerable set of choices of functional motifs since the self-assembling backbone sequence show strong propensity to self-assemble and is less perturbed by functionalization than, for example, peptide amphiphiles^48^ and Fmoc-modified short SAPs^49^, where overall peptide length/polarity affect the stability and the final self-aggregated structure. Also, provided that the same self-assembling backbone is used, RADA16-like peptides allow for the concurrent exposure of a number of different functional motifs within the same scaffold: this is useful to address, for example, pathologies with a complex set of biological pathways involved such as spinal cord regeneration^26^. In respect to coassembling peptides (e.g., LKLK12 and LDLD12),^36^ RADA16-like peptides are neutral at neutral pH and require a shift of solvent pH to trigger their gelation, while the former just need the copresence of both species. Also, in respect to BMHP1-derived peptides, capable of forming complex and hierarchically self-assembled nano- and microstructures^50^, RADA6-like SAPs usually give rise to flat cross-beta fibers, occasionally paired together: this can allow a better control of scaffold properties (i.e., porosity, stiffness, etc.). Like all other SAPs, RADA16 show fragile behavior and limited mechanical properties narrowing their field of application to soft or medium-soft tissues, however this can be overrun via chemical cross-linking^19,20^. Lastly, again in respect to BMHP1-derived SAPs, RADA16-like SAPs do not show significant self-healing properties, which may be detrimental for some regenerative medicine applications.

## Solving manufacture bottlenecks

One of the key issues to be solved for SAP nanofiber scaffold commercialization is affordable scale-up production. For human use, GMP must be followed and be compliant with the relevant requirements of regulatory agencies including ubiquitous quality control requirements through all steps of the product’s development, manufacturing, warehousing, marketing, sales or distribution, after sales services, workforce and customer training, and operational systems and processes. Despite the unique and commercially appealing properties of SAPs, developing viable and affordable SAP-based products has taken almost a decade. Because in the early 2000s peptide production was still too expensive and complicated for initial small-scale industrial production, chemical peptide synthesis turned out to be most efficient. Chemical peptide synthesis also yields a well-characterized, pure product that can be produced with high enough batch-to-batch consistency for medical use.

The high cost of SAPs production narrowed early on any possible applications to the health industry and use in human medicine. SAPs promise potential success in tissue regeneration, sustained drug delivery, and surgical applications, such as stasis of body fluids or reduction of adhesions after surgery. A further advantage of synthetic SAPs is that regulatory authorities may accept them as medical devices, thereby allowing development of products in less time and with less capital. However, the production of small or lab-scale quantities of SAPs for nonhuman use can be achieved by conventional desktop peptide synthesizer and purified by a laboratory HPLC.

SAP manufacturing methods have evolved over the last two decades, going from fixed, dedicated, investment-heavy productions lines to conventional but modular production assemblies, to low-cost, single batch micro production setups. For research purposes only, PuraMatrix^®^ (RADA16) is available in non-GMP grade from various vendors, including 3-D Matrix and others. The same applies to other research purpose polymeric systems which are indeed less expensive to manufacture. Again, compounds destined for medical purposes are subject to strict regulatory and quality control requirements that drive their cost up. In fact, it is not unusual that the chemical production cost alone may end up being lower than sum of all other costs involved in the production of a medical device product.

## Working with regulatory agencies

For any products derived from nanotechnology to be successfully commercialized as medical products, it is critical to work closely with regulatory agencies. Although slowly converging over the last two decades, the regulatory environments among the largest health markets, including the USA, Japan, and Europe, remained different until very recently, at least for medical devices. Simplified market approval of medical devices in any of the world’s larger markets is based on regulatory compliance with relevant laws, on demonstrated safety and performance in human use as evidenced by biosafety studies and clinical trials, and on a quality management system that monitors the product’s R&D, GMP manufacturing, marketing, sales, and use. Interestingly, while market approval of SAPs was easier to achieve in Europe than in the USA until a few years ago, market approval in Europe today has become difficult and slower. This is the result of three linked developments: the stricter European Medical Device Regulations (MDR) replacing the simpler and obsolete Medical Device Directives (93/42/EEC) on May 26, 2021; harmonized quality management guidelines ISO13485:2016 replacing the simpler ISO13485:2012; and over half of all medical device-assessing notified bodies—the organizations mandated by member states of the EU to assess the conformity of certain products before being placed on EU markets (CE-mark)—disappearing as a result of these changes, whether for economic or operational reasons or because they were unable to become certified under the new MDR.

The number of market approval requests did however not decrease as much, and was led partially by the disappearance of small medical device companies that could no longer cope with the new regulations. Overall, it is expected that new SAP-based medical products will start to appear increasingly in the U.S. market already this year, while SAPs have been sold as medical devices on the European markets (e.g., PuraStat^®^) since 2015. As a consequence of these changes, regulatory and quality management aspects of medical devices must be planned even earlier, at the research stage before product development is being considered. As in good design practices, the design of the product should be reverse engineered starting from customer and user input while being continually subject to the quality management system. The product needs to be designed for manufacturing and here, too, quality management is intrinsically part of this process.

SAPs have the advantage that as long as they are inert and do not show distinct biologic activity, they may be regulated as medical devices in most jurisdictions. Medical devices may be approved by the USFDA via a premarket approval process (PMA; Code of Federal Regulations 21 CFR Section 814) or a premarket notification 510(k) (21 CFR 807). Through a PMA, a medical device is demonstrated to be safe and effective for the patient or end user. PMAs require substantial clinical trials. According to the FDA, the goal of a 510(k) instead is “to demonstrate that the device to be marketed is as safe and effective, that is, substantially equivalent, to a legally marketed device.” The need for clinical trials is usually reduced and approval takes less time and is less expensive. In many cases, a 510(k) is a more desired path to commercialization than a PMA process.

## The future of SAPs

Since the initial discovery of SAPs, several clinical applications have been brought to the market. The SAP material continues to have great potential to be exploited into beneficial products. We have only scratched the surface of the possibilities for development. The coming decades promise to show the fulfillment of the potential of this class of materials.

## Methods

### Designer peptide synthesis and scaffold preparation

All self-assembling peptides were dissolved in distilled sterile water (GIBCO) at a final concentration of 1% (v/w) (10 mg/ml) and sonicated for 30 min before use.

### Cell cultures

Neural precursor cultures are established and expanded as previously described. Briefly, neural precursors isolated from the subventricular zone of 8-week-old CD-1 albino mice striata, at passage 10, were used. Cell proliferation was performed in NS-A serum-free medium (Euroclone, Irvine, UK), in the presence of basic fibroblast growth factor (βFGF from PeproTech, Rocky Hill, NJ) and epidermal growth factor (EGF from PeproTech) at final concentrations of 10 and 20 ng/ml. The medium without growth factors was used as a basal medium. Bulk cultures were generated by mechanically dissociating neurospheres and plating cells in untreated flasks at the appropriate density (1 × 104 cells/cm^2^) every 4–5 days in the same growth medium. Cell counting and viability was performed at every passage, using trypan blue exclusion.

### Cell culture seeding

Neural precursor cultures were established and expanded as described previously. In the case of adhesion and differentiation tests, cell seeding (at a concentration of 2–8 × 104 cells/cm^2^) was performed 2 days after the last mechanical dissociation in order to seed the maximum percentage of stem cells. Cells were seeded on the top surface of each assembled scaffold, where they were able to settle into the nanofiber matrices. Over time cells penetrate the self-assembled layer

In the case of SEM imaging, cells were acutely mixed with 8 μl of aqueous gel solution at a final concentration of 5–8 × 103 cells/μl in a total final volume of 10 μl per sample. Self-assembling was then initiated by adding basal medium slowly and placing seeded scaffolds mounted on copper grids (Ted Pella Inc.) at +37 °C for 30 min. Cells were thus already embedded in the matrices.

For both adhesion and differentiation tests and SEM imaging, cells were cultured with basal medium supplemented with βFGF (10 ng/ml), added to enhance neuronal progeny differentiation. After 3 days, the medium was shifted to a medium containing leukemia inhibitory factor (LIF, Chemicon) (20 ng/ml) and brain-derived neurotrophic factor (BDNF, Peprotech) (20 ng/ml) to pursue the neuronal and glial population maturation in NSC progeny. Cells were fed every 3 days with the same fresh culture medium.

### Cell proliferation assay

MTT (3-(4,5-dimethylthiazol-2yl)-2,5 diphenyl tetrazolium bromide) was prepared in a 5 mg/ml stock solution in PBS, then added to the culture medium in a ratio of 1:100. After 1-h incubation at +37 °C, the MTT solution was removed and the insoluble formazans crystals were dissolved by soaking scaffolds and cells for 15 min in 250 μl of dimethylsulfoxide. The absorbance was measured by using a Vmax microplate reader (Molecular Devices, Sunnyvale, CA) at a wavelength of 550 nm.

To assess the viability of NSCs seeded on scaffolds made of various peptides, a quantitative method, MTT assay (Sigma), was used. Four independent experiments comprising three replicates each were performed. For this viability test, the direct proportional linearity between the optical density and the viability/metabolic activity of the cell populations was assessed from verifying the linearity of five different standard curves at six increasing cell concentrations, ranging from 5 × 103 to 5 × 105 cells/well. Results are expressed as percent increase in cell population from the population seeded on day 1.

Cell viability and differentiation assay tests were conducted by pouring aqueous solution of functionalized SAPs hydrogel (30 μl per well) so as to evenly cover the bottom surface of each well (~30 μm gel layer thickness) of 96 multiwell plates (BD Biosciences). In the case of SEM imaging both for Matrigel and the peptide hydrogel, the total amount of biomaterial was reduced to 10 μl. The experimental protocol included control tests conducted with nonfunctionalized RADA16 (negative control) and Matrigel coating (positive control). RADA16 and other functionalized peptides hydrogel: poured 30 μl/well of a 1% (w/v) distilled sterile water solution, followed by slow addition of 200 μl/well of basal medium. Allowed to self-assemble at 37 °C for 30 min and rinsed once with control medium to wash away any residual acid residues remaining from peptide synthesis. Matrigel GF-reduced (from EHS sarcoma, BD Biosciences): diluted 1:100 in basal medium, poured at 100 μl/well, 30 min incubation at 37 °C, then rinsed with basal medium. In the case of SEM imaging, no dilution was adopted in order to guarantee the necessary stiffness for a three-dimensional scaffold.

### Immunocytochemistry

Neuronal and glial differentiation was assessed by double and single immunostaining with lineage-specific antibodies: anti-Nestin (1:150, Chemicon) for progenitor cells, rabbit anti-β-tubulin (1:500, Covance) for neurons, and mouse anti-glial fibrillary acidic protein (1:200, Chemicon) for astrocytes. Primary antibodies were then stained with secondary ALEXA 488 goat anti-mouse (1:1000, Molecular Probes) and CY3 AffiniPure F(ab′)2 anti-rabbit IgG antibodies (1:100, Jackson Immuno Research). Cell nuclei were counterstained with DAPI (Molecular Probes). The samples were then examined by inverted fluorescence microscope. Quantitative analyses were performed by counting 100–300 cells for each of ten nonoverlapping (and randomly chosen) fields. Four independent experiments comprising two replicates each were performed.

### SEM sample preparation

Samples were prepared as described previously. Briefly, the matrices were soaked in 5% glutaraldehyde at 4 °C for 2 h, followed by washing in MilliQ water and slow sequential dehydration steps in 10% increments of ethanol for 5 min each. Samples were then placed in pressurized liquid CO_2_/siphon for 1 h using a CO_2_ critical point dryer (Tousims). Scaffolds were next sputter-coated with gold-palladium particles (~10 nm gold coating thickness), mounted on a copper grid and examined.

### SEM sample preparation and imaging

After seeding NSCs within the self-assembled scaffolds as previously described (cell seeding section), cells were cultured for 7 and 14 days in NSC basal medium. The peptide scaffold was prepared for SEM: the matrices were soaked in 5% glutaraldehyde at 4 °C for 2 h, followed by washing in MilliQ water and slow sequential dehydration steps in 10% increments of ethanol for 5 min each. Samples were then placed in pressurized liquid CO_2_/siphon for 1 h using a CO_2_ critical point dryer (Tousims). Scaffolds were next sputter-coated with gold-palladium particles (~10 nm gold coating thickness), mounted on a copper grid, and examined using a JOEL JSM 6060 SEM at ×2000–100,000 magnification, 6 KV acceleration voltage, 29–32 spot size, and 12 mm electronic working distance.

### In vitro neural stem cell cultures

3D scaffolds were self-assembled on cell culture inserts, PET membrane, 1.0 μm pore size (BD Biosciences). SAPs were dissolved in sterile distilled water (GIBCO) to double the desired concentration the day before cell plating. On the day of scaffold preparation, 12 μl of each SAP was diluted with 12 μl of glucose 8%, to balance cellular osmolarity, to the final desired concentration. A volume of 24 μl of each SAP was gently mixed with 8 μl of culture medium containing a cell density of 4000 cells/μl, in a total of 3.2 × 10^4^ murine neural stem cells. The mixture was placed on the insert membrane, subsequently placed in the well containing basal cell culture medium supplemented with 20 ng/ml LIF (Chemicon), and 20 ng/ml BDNF (Peprotech) to foster cell differentiation and allowed to self-assemble at 37 °C. The culture medium was replaced every 3 days. Cells were subsequently labeled with live/dead cell assay (Molecular Probes) following the provided protocol in the datasheet.

### High-density 3D cell seeding

Before seeding, NSCs were separately cultured at density of 10^4^ cells/cm^2^ using serum-free medium in the presence of βFGF (PeproTech) and EGF (PeproTech) at final concentrations of 10 and 20 ng/ml, respectively. Neurospheres were mechanically dissociated every 10 days in the same growth medium. Briefly, peptide hydrogel (dissolved 1% w/v in dH_2_O, GIBCO) was mixed with sucrose, NaOH, and culture medium containing a cell density of 4.5 × 104 cells/ml. A droplet of 40 ml was placed in a 24-well and serum-free medium supplemented with bFGF (20 ng/ml) was added to generate 3D scaffolds. At 2 days in vitro, medium was shifted with a basal medium supplemented with LIF (20 ng/ml, Chemicon) and BDNF (20 ng/ml, PeproTech). Fresh medium was replaced every 3 days.

### Hydrogel implants for nervous regeneration

SAPs were dissolved in sterile distilled water (GIBCO) at 1% (w/v) concentration. Depending on the tested regenerative medicine application, a dedicated injury has to be imposed to the animal in order to provide space for scaffold placement. SAP can be injected or preassembled into electrospun microchannels (made of synthetic polymers such PCL and PLGA or of cross-linked SAPs) before placement. In the latter case, an additional dose of pure SAP into the implant site is recommended in order to better fill the gaps among the channels and provide a continuum scaffold filling the site of injury. Scaffolds can be later examined, after animal perfusion, with standard histology techniques.

## Data Availability

The datasets generated during and/or analyzed during these studies are available from the corresponding author on reasonable request.
